# Translating the 2023 Malaysian Heart Failure Clinical Practice Guidelines (5th Edition) into primary care practice

**DOI:** 10.51866/cpg.1108

**Published:** 2026-04-28

**Authors:** Say Yee Loo, Ping Foo Wong, Tiong Lim Low, Yean Yee Loke, Lee Boon Kiew, Mohd Shawal Faizal Mohamad

**Affiliations:** 1 Department of Family Medicine, Faculty of Medicine, Universiti Kebangsaan Malaysia, Jalan Yaacob Latif, Bandar Tun Razak, Cheras, Kuala Lumpur, Malaysia.; 2 Klink Kesihatan Cheras, No. 14, Jalan Yaacob Latif, Bandar Tun Razak, Cheras, Kuala Lumpur, Malaysia.; 3 Klinik Kesihatan Rawang, Jalan Rawang Perdana, Taman Rawang Perdana, Rawang, Selangor, Malaysia.; 4 Klinik Kesihatan Batu Muda, No. 7, Jalan 3/124, Kampung Batu Muda, Kuala Lumpur, Malaysia.; 5 Klinik Kesihatan Tudan, Jalan Tudan Permyjaya, Miri, Sarawak, Malaysia.; 6 Department of Medicine, Faculty of Medicine, Universiti Kebangsaan Malaysia, Jalan Yaacob Latif, Bandar Tun Razak, Cheras, Kuala Lumpur, Malaysia.

**Keywords:** Heart failure, Guideline, Malaysia, Primary care physicians, Guideline

## Abstract

Heart failure (HF) is a major public health concern in Malaysia, with increasing prevalence and significant morbidity and mortality. The 2023 Malaysian HF Clinical Practice Guidelines (CPGs) (5th Edition) provide updated, evidence-based recommendations tailored for local practice, particularly in primary care, where long-term management often occurs. Compared with international guidelines, the Malaysian CPGs adopt age-specific natriuretic peptide (NP) thresholds to improve diagnostic specificity while acknowledging that diagnosis may still depend primarily on clinical assessment supported by electrocardiogram (ECG) and chest radiography in resource-limited settings. This article provides a narrative educational review that summarises the core recommendations of the Malaysian HF CPGs, compares them with international guidelines and illustrates their application in primary care through two case vignettes representing different HF classifications. We aim to provide practical application of Malaysian CPG recommendations in primary care, emphasising the need for primary care physicians to initiate foundational HF therapies early and titrate them to target or maximally tolerated doses while ensuring their continuation in the long term to prevent relapse and preserve ventricular function. In addition, the availability of NP testing, diastology studies and structured titration protocols in primary care would strengthen early diagnosis and systematic optimisation of therapy, ultimately improving outcomes for patients with HF.

## Introduction

Heart failure (HF) is a clinical syndrome caused by structural and/or functional abnormalities of the heart, confirmed by elevated natriuretic peptide (NP) levels and/or objective evidence of cardiogenic pulmonary or systemic congestion. In line with the 2021 Universal Definition and Classification of HF, which is a collaborative effort between American, European, Japanese and other international societies, the Malaysian clinical practice guidelines (CPGs) also recommend classifying HF according to left ventricular ejection fraction (LVEF) phenotype categories, including HF with reduced ejection fraction (HFrEF), HF with mildly reduced ejection fraction (HFmrEF) and HF with preserved ejection fraction (HFpEF), and staging the condition to at risk for HF (stage A), pre-HF (stage B), HF (stage C) and advanced HF (stage D).^1^

HF is a growing public health concern in Malaysia, with one of the highest prevalence rates in Southeast Asia. The prevalence increased from 7.7% in 1990 to 721 cases per 100,000 population in 2017.^2^ The MY-HF Registry showed that patients were predominantly men, with a mean age of 60.2 years, often presenting with advanced symptoms, such as New York Heart Association (NYHA) class III or IV, and largely driven by ischaemic heart disease.^3^

Globally, HF affects approximately 64 million people, and this figure is expected to increase with an ageing population, contributing to high morbidity and mortality and substantial healthcare burden.^4^ This narrative review aimed at helping primary care physicians to apply the 2023 Malaysian HF CPGs through practical comparison with the European Society of Cardiology (ESC)/American Heart Association (AHA) guidelines and two illustrative case vignettes.

**Why are CPGs compared, and what are the key recommendations of the Malaysian CPGs?**

In Malaysia, much of the long-term care for HF is provided by non-cardiologists, particularly family medicine physicians. Therefore, it is crucial to ensure that the Malaysian CPGs for HF remain practical and readily applicable in the primary care setting. While international guidelines (ESC/AHA guidelines) and the Malaysian CPGs share the same goals, including correct diagnosis, LVEF phenotype classification, rapid initiation and optimisation of evidence-based treatment and prevention of hospital readmission, they differ in their emphasis of certain recommendations due to local adaptation for resources and practical implementation pathways.^5-8^

In this review, we examined the 2023 Malaysian HF CPGs, the 2021 ESC HF guidelines (including the 2023 focused update) and the 2022 AHA/ACC/HFSA HF guideline, focusing on a) definition and classification, b) diagnostic thresholds and c) pharmacological management of HFrEF/HFmrEF/HFpEF.

- *Core definition and classification*
  All three guidelines adopt the 2021 Universal Definition of HF, which requires symptoms and/or signs to be supported by objective evidence of cardiac dysfunction. They also use the same ejection fraction (EF)-based phenotypes as a framework for classification.
- *Diagnostic thresholds*
  All three guidelines emphasise an initial NP measurement, followed by echocardiography and targeted investigation. In the non-acute setting, a B-type natriuretic peptide (BNP) level of ≥35 ng/L or an N-terminal pro-B-type natriuretic peptide (NT-proBNP) level of ≥125 ng/L is used, while in the acute setting, the threshold is a BNP level of ≥100 ng/L or an NT- proBNP level of ≥300 ng/L.
  The Malaysian CPGs highlight a more specific approach to identify possible HF in emergency settings, recommending a BNP level of >400 ng/L or an NT-proBNP level of >450 ng/L for patients aged under 50 years, >900 ng/L for those aged 50–75 years and >1800 ng/L for those aged over 75 years. These higher thresholds aim to improve diagnostic specificity. However, NP testing is not widely available in Malaysian primary care, creating a barrier to early diagnosis. In resource-limited settings, a pragmatic approach is often needed, relying on clinical assessment, supported by ECG, chest radiography and, if accessible, NT-proBNP testing as well as echocardiography to improve diagnostic accuracy.^9^
- *Pharmacological management of HFrEF/HFmrEF/HFpEF*
  All three guidelines agree on the four foundational drug classes for HFrEF, including renin– angiotensin system (RAS) blockers/angiotensin receptor neprilysin inhibitors (ARNis), beta blockers, mineralocorticoid receptor antagonists (MRAs) and sodium–glucose cotransporter 2 inhibitors (SGLT2-is). For HFmrEF and HFpEF, both the ESC and AHA strongly recommend the use of SGLT2-is to reduce the risk of HF-related hospitalisation and cardiovascular death. The Malaysian CPGs adopt these recommendations but tailor them to the local context, taking into account drug availability, cost and the need for appropriate monitoring. Table 1 presents the comparison of HF management across the major guidelines.^5-8^

**Table 1:** Comparison of HF management across the major guidelines.

Aspect	2023 Malaysian HF CPGs		2021 ESC HF guidelines (including the 2023 focused update)		2022 AHA/ACC/HFSA HF guideline	
Foundational medication: RAS blockers/ARNi Beta blockers SGLT2-i MRA	HFrEF	All four foundational medications are recommended. Diuretics for volume overload. SGLT2-i is recommended, as it demonstrated effectiveness regardless of diabetic status.	HFrEF	All four foundational medications and diuretics for fluid retention are recommended (Class 1).	HFrEF	All four foundational medications and diuretics are recommended (Class 1). ARNI is preferred over ACEi/ARB.
	HFmrEF	SGLT2-i is recommended. Diuretics for volume overload. Others can be considered.	HFmrEF	SGLT2-i and diuretics for fluid retention are recommended (Class 1). Others: class Ilb recommendation.	HFmrEF	Diuretics, as needed, are recommended (Class 1). SGLT2-i can be beneficial (Class 2 a). Others can be considered (Class 2b).
	HFpEF	Diuretics for volume overload. SGLT2-i is recommended. ARNI/ARB/MRA can be considered. Beta blockers have limited evidence of benefit.	HFpEF	SGLT2-i and diuretics for fluid retention are recommended (Class 1).	HFpEF	Diuretics, as needed, are recommended (Class 1). SGLT2-i can be beneficial (Class 2 a). ARNI/ARB/MRA can be considered (Class 2b).
Initiation setting	As inpatient	, even if started at low doses.	As inpatient with rapid up titration within 48 hours before hospital discharge to reach at least half of the target doses of recommended medications.		As inpatient after clinical stability is achieved.	
Titration timeline	Gradual outpatient up titration to maximally tolerated dose within 12 weeks of discharge, with adjustment based on patients’ haemodynamic stability and renal function.		Titration to full target doses attempted within 2 weeks after discharge with appropriate safety monitoring.		Titration and optimisation may be performed as frequently as every 1—2 weeks and should be individualised to patients’ clinical status.	

**HF:** Heart failure; **CPGs:** Clinical Practice Guidelines; **ESC:** European Society of Cardiology; **AHA/ACC/HFSA:** American Heart Association / American College of Cardiology / Heart Failure Society of America; **HFrEF:** Heart Failure with reduced Ejection Fraction; **HFmrEF:** Heart Failure with mildly reduced Ejection Fraction; **HFpEF:** Heart Failure with preserved Ejection Fraction; **RAS:** Renin-Angiotensin System; **ARNi:** Angiotensin Receptor- Neprilysin Inhibitor; **ACEi:** Angiotensin-Converting Enzyme inhibitor; **ARB:** Angiotensin II Receptor Blocker; **MRA:** Mineralocorticoid Receptor Antagonist; **SGLT2-i:** Sodium-Glucose Cotransporter-2 inhibitor.

While referral for cardiology consultation is important, initiation and optimisation of HF therapy should not be delayed. Early treatment in primary care provides crucial cardiovascular protection while awaiting specialist review.^9^ Increasing referrals indicate the challenges encountered by primary care physicians in navigating the evolving HF care, including new medications and advanced interventions.^9,10^

**How can the CPGs be applied in primary care?**

In practice, implementation in primary care may vary depending on local access to natriuretic peptide testing, echocardiography, medication availability, laboratory monitoring, and timely referral pathways. Therefore, while primary care physicians should be encouraged to initiate and optimise evidence-based HF therapies whenever feasible, management should also be individualised according to the resources and shared-care support available in each setting. Herein, we present two common cases seen in primary care and the application of the 2023 HF CPGs for each of them.^7^

### Case 1

Ms TSK was a 53-year-old woman attending her regular follow-up at the primary care clinic, during which she reported worsening shortness of breath over the past month. Initially experiencing breathlessness only after prolonged treadmill jogging, she then noted difficulty breathing even with mild exertion and developed orthopnoea, needing two pillows to sleep comfortably. However, she denied episodes of sudden breathlessness at night (no paroxysmal nocturnal dyspnoea). She also described worsening swelling of both ankles, noticeable throughout the day, and a recent unintended weight gain from 89 kg to 94 kg within 1 month. She denied chest pain, palpitations, dizziness or syncope.

Ms TSK had a history of hypertension for more than 10 years. Despite being on treatment, her home blood pressure readings were suboptimally controlled, ranging 140–150/90–100 mmHg. Other comorbidities included dyslipidaemia, obesity class II with body mass index (BMI) of 35.8 kg/m2 and prediabetes (HbA1c level of 6.2%). Her medications were amlodipine 5 mg once daily (OD), perindopril 8 mg OD, bisoprolol 5 mg OD and atorvastatin 20 mg OD.

On physical examination, her blood pressure was 171/103 mmHg (rechecked reading: 179/89 mmHg), with a heart rate of 94 bpm and an oxygen saturation of 98% on room air. She appeared comfortable at rest with warm peripheries and good peripheral pulses. Her cardiovascular and respiratory examination findings were unremarkable, but bilateral pitting oedema was observed up to the mid-shin level.

Ms TSK was clinically suspected to have HF based on her symptoms and signs.

***Q1: What diagnostic approach should be taken in Ms TSK when HF is suspected?***

In Ms TSK’s case, the suspicion of HF arose during a routine primary care follow-up when she reported worsening shortness of breath, reduced exercise tolerance, orthopnoea and new-onset bilateral ankle oedema. These symptoms alone raised clinical concern, particularly in the context of her multiple cardiovascular risk factors, including long-standing hypertension and obesity.

According to the national guidelines, HF is defined as a clinical syndrome caused by structural or physiological abnormalities of the heart, leading to impaired ability to meet metabolic demands or doing so only at higher-than-normal filling pressures. This diagnosis is supported by objective findings such as elevated NP levels or signs of fluid overload.

As frequently conducted in primary care, the initial step was focused history-taking and physical examination, which remain the cornerstone of HF diagnosis. In Ms TSK’s case, the clinical picture was consistent with stage C HF. She reported no chest pain or palpitations, and her vital signs were stable aside from persistently elevated blood pressure. On examination, there were no pulmonary crepitations or elevated JVP, but her symptomatic fluid overload and progressive weight gain over 1 month were concerning enough to pursue further workup.

Given the resource availability in primary care, we proceeded with ECG, which showed left ventricular hypertrophy (LVH), a common consequence of chronic uncontrolled hypertension and a clue towards underlying structural cardiac changes. Chest radiography revealed mild cardiomegaly, further supporting the working diagnosis.

**Figure 1:**
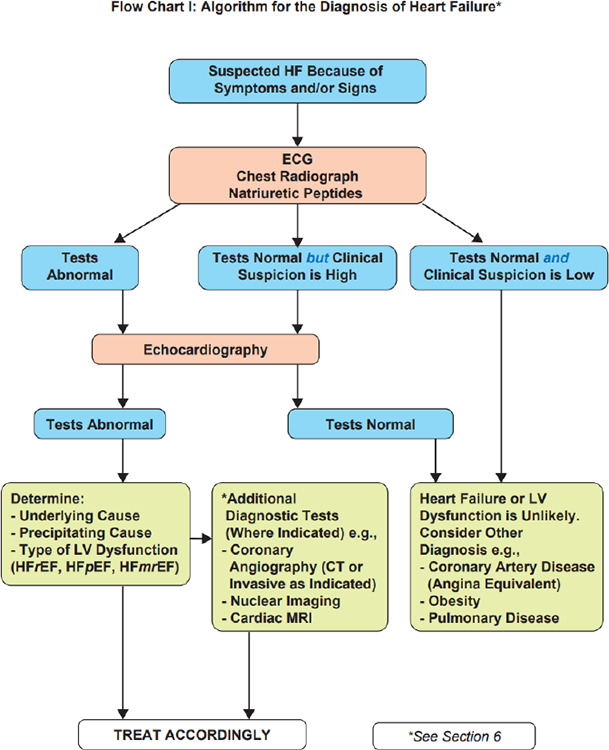
Diagnostic pathway.^7^

Although subtle, this aligned with her symptoms and risk profile. While BNP or NT-proBNP testing is not routinely available in all primary care clinics, it would have been helpful in this case, where a BNP level of <3;5 ng/L or an NT-proBNP level of <1;25 ng/L could rule out HF. However, Ms TSK’s clinical features were suggestive, and even if BNP testing were available, its interpretation must be undertaken with caution in obese individuals such as this patient, as levels may be falsely low. Ultimately, echocardiography remains the gold standard method for confirming the diagnosis of HF (Figure 1).

***Q2: How is HF staged in a patient such as Ms TSK?***

Ms TSK’s HF was classified under stage C, and her symptoms were consistent with NYHA class II. At this point, she required active medical management to prevent further progression and improve her symptoms.

Ms TSK had been in stage A for years, living with several key risk factors for HF, including poorly controlled hypertension, obesity, dyslipidaemia and prediabetes. At that time, she was asymptomatic, and prevention should have been the priority. Tighter blood pressure control, weight reduction, increased physical activity and better risk factor management might have delayed or prevented progression to symptomatic HF.

If identified earlier, her HF may have remained in stage B, where patients are still symptom-free but are on the path towards HF. Recognising and treating patients during this ‘silent’ phase are crucial, as they allow for early intervention before symptoms begin. Her current presentation serves as a reminder that HF often evolves gradually, and the earlier the intervention, the greater the likelihood of altering its course.

***Case 1 (continued)***

During the same visit, Ms TSK was started on oral furosemide 40 mg OD to relieve symptoms of fluid overload, particularly her bilateral pedal oedema. In addition, oral spironolactone 12.5 mg OD was initiated to support blood pressure control and for its cardiovascular benefits in patients with suspected HF. She was scheduled for close follow-up in primary care to monitor symptom resolution and optimise blood pressure.

She was subsequently evaluated at the cardiac centre, where her NT-proBNP level was 125 ng/L. Transthoracic echocardiography showed a normal EF of 56% and borderline LVH. There was evidence of impaired relaxation consistent with diastolic dysfunction. The left atrial, right ventricular and right atrial sizes were normal, with only mild septal LVH seen. The estimated pulmonary artery systolic pressure was normal at 24 mmHg, and no significant valvular abnormalities were detected.

Given the preserved EF and absence of overt wall motion abnormalities or segmental changes, no immediate testing for myocardial ischaemia was indicated at this stage. Ms TSK denied any chest pain or anginal equivalents, and her functional limitation was consistent with HF symptoms rather than ischaemic burden.

Following the cardiology review, Ms TSK was referred back to primary care for co-management. Her symptoms had improved with the initial introduction of furosemide and spironolactone, and her blood pressure showed better control at follow-up.

***Q3: What classification of HF does Ms TSK have, and how should she be managed in the primary care setting?***

Ms TSK was diagnosed with HFpEF (Table 1). In primary care, her management focused on both symptom control and long-term risk reduction. Following initial improvement in congestion with furosemide, her fluid status was reassessed regularly, and the diuretic dose was tapered to the lowest effective amount to maintain euvolaemia. Simultaneously, spironolactone was continued as part of her antihypertensive regimen, given its added benefit in addressing volume status and blood pressure.

Given her elevated cardiovascular risk and prediabetic status, oral empagliflozin 10 mg OD was initiated. This medication not only supports glycaemic control but also reduces HF-related hospitalisations and improves outcomes in patients with HFpEF. Her blood pressure had since improved with the combined regimen of perindopril, bisoprolol, spironolactone and empagliflozin.

Non-pharmacological care played an equally important role in her long-term management. Ms TSK had remained physically active, maintaining her routine of gym workouts three times a week and combining both aerobic and resistance training. She was referred to a dietitian to support calorie restriction and gradual weight loss, although she declined referral to an obesity clinic. Regular follow-up visits included ongoing education on self-monitoring, particularly recognising early signs of fluid overload such as sudden weight gain, increasing orthopnoea and ankle swelling. She was advised to monitor her daily weight, reduce salt intake and maintain fluid restriction if symptoms recurred (Table 2).

**Table 2:** HF classification.^7^

Ejection Fraction Terminology	LVEF
Heart Failure with Reduced Ejection Fraction (HFrEF)	≤ 40%
Heart Failure with mildly reduced LVEF (HFmrEF)	41-49%
Heart Failure with Preserved Ejection Fraction (HFpEF)	≥ 50%
Heart Failure with Improved Ejection Fraction (HFimpEF)	HF with a baseline LVEF of ≤ 40%, a ≥10-point increase from baseline LVEF following treatment, and a second measurement of LVEF of > 40%.

In primary care, her follow-up focused on optimising medications, ensuring her blood pressure remained well controlled and supporting her lifestyle goals. The aim was to maintain symptom stability, prevent decompensation and reduce the risk of HF-related hospitalisation. With shared care between cardiology and primary care, Ms TSK’s condition remained under close monitoring and holistic management.

### Case 2

Mr Sim was a 50-year-old businessman and an ex-smoker with a history of HFrEF and anterior ST elevation myocardial infarction, for which he underwent primary percutaneous coronary intervention at a private hospital in 2024. He had no history of diabetes or hypertension. An echocardiogram performed in 2024 showed an LVEF of 25%–35% (31.5%), regional wall motion abnormalities, a severely dilated left atrium, a mildly dilated right atrium and structurally normal valves. His ECG demonstrated a sinus rhythm.

During his routine clinic follow-up, his symptoms were consistent with NYHA class II and Canadian Cardiovascular Society (CCS) class I angina. He remained adherent to his medications, which included perindopril 2 mg OD, spironolactone 25 mg OD, furosemide 20 mg OD, atorvastatin 40 mg ON and Cardiprin 100 mg OD.

On examination, his blood pressure was 111/76 mmHg; pulse rate, 72 bpm; and oxygen saturation, 98% on room air. His weight was 101 kg, with a BMI of 35.5 kg/m^2^. There were no clinical signs of cardiac decompensation. Recent laboratory results showed a serum potassium level of 4.2 mmol/L and a creatinine level of 87 pmol/L (eGFR: 94 mL/min/1.73 m^2^).

***Q1: How should Mr Sim be managed in the primary care setting?***

According to the national guidelines, Mr Sim should be started on all four foundational medications for HFrEF, including RAS blocker or ARNi, beta blockers, MRAs and SGLT2-is. In addition to his current treatment with perindopril and spironolactone, bisoprolol 1.25 mg OD was started, while spironolactone was increased to 50 mg OD and perindopril to 4 mg OD.

In line with guideline recommendations, these agents should be uptitrated to the maximally tolerated or target doses as early as possible. As he was clinically euvolaemic, furosemide 20 mg was advised to be taken only when needed. Given these medication adjustments, regular monitoring of renal function and electrolytes is essential.

For non-pharmacological management, Mr Sim was educated on home blood pressure monitoring and recognition of the signs of decompensation, such as rapid weight gain, peripheral oedema, orthopnoea or paroxysmal nocturnal dyspnoea. He was also counselled on ‘dry weight’ management, with instructions to record his daily weight and take furosemide 20 mg OD together with fluid restriction if his weight increased by more than 2 kg within 3 days. He was advised to seek prompt medical attention if his weight increment was accompanied by worsening symptoms or if he failed to respond to these measures.

***Case 2 (continued)***

During review 3 weeks later, Mr Sim reported significant symptomatic improvement, with NYHA class I functional status. His blood pressure was 107/69 mmHg without postural hypotension; pulse rate, 60 bpm; and weight, 101.5 kg. Laboratory results showed stable renal function with no evidence of hyperkalaemia or renal impairment.

***Q2: What is the next step in the long-term management in the primary care setting?***

The next step in the management of HFrEF was to optimise his medical therapy, guided by clinical status, heart rate, blood pressure, fluid status, renal function, electrolyte level and tolerance. Perindopril was uptitrated to 6 mg OD, and empagliflozin 10 mg OD was added to complete the four foundational HF therapies. Bisoprolol was continued at 1.25 mg OD, as the target heart rate of 50–60 bpm had been achieved. Renal function tests were scheduled for repeat monitoring in 2 weeks following these adjustments (Table 3).

**Table 3:** Primary care management of HFpEF.^7^

Intervention	Grades of Recommendation	Levels of Evidence	Comments
LIFESTYLE MEASURES			
Overweight / Obesity	I	B	A caloric restriction diet is feasible and safe and should ideally be combined with exercise. Bariatric surgery in patients with HFpEF and obesity was associated with improved symptoms and reduction in HF hospitalizations.
Exercise Training	I	A	This is safe and improves exercise capacity and quality of life.
IDENTIFYING AND TREATING THE UNDERLYING CAUSE(S) AND CO-MORBIDITIES			
Hypertension		A	Improved BP control has been shown to reduce morbidity and hospitalizations for HF.
Tachyarrhythmias (Persistent or Paroxysmal AF)	IIa	B	**Rate control** with (3-blockers or non-dihydropyridine calcium channel blockers (verapamil, diltiazem) alone or in combination.
	IIa	A	**Rhythm control** in patients with recent onset AF < 1 year duration or paroxysmal AF.
Anti Coagulation		A	To reduce the risk of thromboembolic events.
Others		C	Treat CAD, diabetes, CKD appropriately according to guidelines.
PHARMACOTHERAPY			
Diuretics		C	To relieve congestion.
RAS Blockers	IIb	B	Trial data show a reduction in HF hospitalizations, but no reduction in all-cause or CV mortality in HFpEF. With ARNI, there was a suggestion of benefit in patients with LVEF < 57% (in women benefits of ARNI were sustained up to LVEF 60%, while for men the benefit was restricted to LVEF 45%).
MRA	IIb	B	It may be considered to decrease HF hospitalizations, particularly among patients with LVEF on the lower end of this spectrum.
SGLT2-i	IIa	A	These have been shown to decrease HF hospitalizations and CV mortality. As more trial data becomes evident, the grading may move upwards to I, A.
ß-Blockers			No good data to show that (3-blockers are beneficial in the treatment of HFpEF. Often prescribed for treatment of co-morbidities.

During follow-up in 2025, a repeat echocardiogram demonstrated improved left ventricular function, with LVEF rising from 31.5% to 43.6% (35%–45%). Clinically, he remained stable in NYHA class I and CCS class I. All four foundational HF medications had been successfully titrated to target doses, reflecting optimal medical therapy in accordance with national guideline recommendations (Table 4). Beyond foundational therapy, additional treatment may be indicated in selected patients, including ARNi, digoxin, ivabradine, implantable cardioverter- defibrillators, cardiac resynchronisation therapy and pacemakers. However, these options were not pursued in this case, as they were not clinically relevant.

**Table 4:** Titration plan for HFrEF.^7^

Intervention	Grades of Recommendation	Levels of Evidence	Comments
INDICATED FOR FLUID RETENTION IN NYHA II - IV			
Diuretics	I	B	No randomized trial to show improvement in survival.
INDICATED IN ALL PATIENTS			
ACE-I	I	A	Improves survival and delays progression in all classes of HF.
ARB	I	A	In ACE-I intolerant patients.
ARNI (Instead of ACE-I)	I	B	Improves survival and delays progression in all classes of HF when compared to ACE-I.
β-Blockers	I	A	Improves survival and delays progression in all classes of HF.
SGLT2-i	I	A	Improves survival and delays progression in all classes of HF.
Mineralocorticoid Receptor Antagonists	I	A	Improves survival and reduces hospitalizations in moderate to severe HF and in post MI patients with mild HF.
IN ADDITION TO THE ABOVE, THE FOLLOWING ARE INDICATED IN SELECTED PATIENTS			
ARB (instead of ACE-I)	I	B	In patients post MI and LVEF < 40%, Valsartan was shown to be comparable to captopril.
Digoxin	I	B	In patients with HF and AF
	IIa	B	No effect on survival. Reduces hospitalizations when added to optimal medical therapy.
Ivabradine	IIa	B	Reduces hospitalizations when added to optimal medical therapy in patients in sinus rhythm and heart rate > 70bpm
ICD (Implantable Cardioverter Defibrillator)	I	A	Improves survival in patients with resuscitated cardiac arrest, VF, or sustained VT
	I	A	Improves survival in patients > 40 days post MI, LVEF < 30%, with non-sustained VT **AND** inducible sustained VT or VF during an EP study and on optimal medical treatment, and in NYHA II or III
	I	A	Improves survival in patients with prior MI and > 40 days post MI and 3 months after revascularization, LVEF < 35% and NYHA class II-III
	I	B	Improves survival in patients (no prior MI), LVEF < 35%, on optimal medical treatment, and in NYHA II or III
INDICATED FOR FLUID RETENTION IN NYHA II - IV			
Diuretics		B	No randomized trial to show improvement in survival.
INDICATED IN ALL PATIENTS			
ACE-I		A	Improves survival and delays progression in all classes of HF.
ARB		A	In ACE-I intolerant patients.
ARNI (Instead of ACE-I)		B	Improves survival and delays progression in all classes of HF when compared to ACE-I.
β-Blockers		A	Improves survival and delays progression in all classes of HF.
SGLT2-i		A	Improves survival and delays progression in all classes of HF.
Mineralocorticoid Receptor Antagonists		A	Improves survival and reduces hospitalizations in moderate to severe HF and in post MI patients with mild HF.
IN ADDITION TO THE ABOVE, THE FOLLOWING ARE INDICATED IN SELECTED PATIENTS			
ARB (instead of ACE-I)		B	In patients post MI and LVEF < 40%, Valsartan was shown to be comparable to captopril.
Digoxin		B	In patients with HF and AF
	Ila	B	No effect on survival. Reduces hospitalizations when added to optimal medical therapy.
Ivabradine	Ila	B	Reduces hospitalizations when added to optimal medical therapy in patients in sinus rhythm and heart rate ≥ 70bpm
ICD (Implantable Cardioverter Defibrillator)		A	Improves survival in patients with resuscitated cardiac arrest, VF, or sustained VT
		A	Improves survival in patients > 40 days post MI, LVEF ≤ 30%, with non-sustained VT **AND** inducible sustained VT or VF during an EP study and on optimal medical treatment, and in NYHA II or III
		A	Improves survival in patients with prior MI and > 40 days post MI and 3 months after revascularization, LVEF ≤ 35% and NYHA class II-III
	I	B	Improves survival in patients (no prior MI), LVEF ≤ 35%, on optimal medical treatment, and in NYHA II or III
CRT (Cardiac Resynchronisation therapy)	I	A	Improves survival in patients having **all of the following:** sinus rhythm, LVEF ≤ 35%, LBBB and QRS duration on resting 12-lead ECG: ≥ 150ms
	IIa	B	≥ 120-149ms
Pacemaker	I	A	For significant symptomatic bradyarrhythmias, trifascicular BBB, third- or high-degree AV blocks.

## Conclusion

The 2023 Malaysian HF CPGs provide a practical and comprehensive framework for translating evidence-based care into primary care practice. Early diagnosis remains essential, beginning with careful history-taking and examination, supported by simple investigations such as ECG and chest radiography and complemented by NP testing and echocardiography, where available. Referral to cardiology should not delay initiation of treatment, as timely therapy in primary care offers crucial cardiovascular protection. Primary care physicians should feel empowered to initiate and titrate all four foundational therapies, rather than waiting for cardiology review.

For patients with HFrEF, the cornerstone ofmanagement is the early initiation ofall four foundational therapies, including RAS blockers, beta blockers, MRAs and SGLT2-is, with careful uptitration to target or maximally tolerated doses. For HFpEF, SGLT2-is now represent the only therapy proven to reduce hospitalisations and cardiovascular death. These treatments must be continued in the long term, even in patients who show recovery of EF, to prevent relapse and deterioration.

Beyond pharmacological therapy, structured protocols in primary care such as standardised titration pathways, access to NP testing and diastology assessment are essential to improve HF outcomes in Malaysia. With these effective strategies, primary care physicians play a crucial role in the early recognition, initiation and long-term optimisation of HF therapy alongside tertiary care centre colleagues to improve patient outcomes and reduce the overall healthcare burden.
